# Deep learning for early detection of cerebral small vessel disease using self-supervised graph embeddings and retinal image analysis

**DOI:** 10.1038/s41598-026-48421-6

**Published:** 2026-04-15

**Authors:** S. Nandhini, K. Vanitha

**Affiliations:** Department of Computer Science and Engineering, Faculty of Engineering, Karpagam Academy of Higher Education, Coimbatore, Tamil Nadu India

**Keywords:** Neural, Transformers, CSVD, Neuro-ophthalmic, Blood vessels, Eye diseases, Cognitive neuroscience, Diseases of the nervous system

## Abstract

The primary driver or cause of cognitive decline and stroke is Cerebral Small Vessel Disease (CSVD), which currently requires neuroimaging tests, which are expensive to obtain and inaccessible in standard clinical settings. Low-cost retinal imaging techniques offer non-invasive assessments that mirror the condition of the brain’s small blood vessels (cerebral microvasculature). State-of-the-art diagnostic methods currently have no accessible, non-invasive, or cost-effective solution to identify CSVD at its earliest stages through visual assessment of retinal biomarkers. This study presents the Retino-Neuro Vision Transformer (RNV-T) framework as a proposed method to detect CSVD utilizing multimodality retinal imaging. The model system comprises five fundamental phases, beginning with Local Vascular Extraction (LVE), followed by Global Transformer-based Encoding (GTE), then proceeding to Graph-Based Relational Learning through Graph-based Convolutional Attention Network (G-CAN) before implementing Local-Global Attention Fusion (LGAF) as well as Optimized training procedures to obtain precise micro-vascular abnormality detection. The diagnostic performance of this model reaches 98.8% accuracy and shows 97.4% sensitivity along with 98.1% specificity, surpassing previous detection approaches. This diagnostic system represents a major leap forward in neuro-ophthalmic care because it enables early prediction of CSVD while expanding medical accessibility through retinal scans that are easy to conduct.

**Supplementary Information:**

The online version contains supplementary material available at 10.1038/s41598-026-48421-6.

## Introduction

The worldwide prevalence of Cerebral Small Vessel Disease (CSVD) as a major stroke and cognitive decline cause makes it difficult to identify until compatible injury develops^1,2^. Several contemporary approaches to diagnosing CSVD involve magnetic resonance imaging (MRI), but these methods are both expensive and challenging to manage, and they are inaccessible in basic medical care centers^3^. Multiple modern neuro-ophthalmic studies stress that the retina shows a reflective resemblance to cerebrovascular health because it shares anatomical and physiological features with cerebral microvasculature^4^. The correlation between retinal imaging and cerebral microvasculature makes it an effective non-invasive diagnostic method using equipment such as Optical Coherence Tomography Angiography (OCTA), Fundus Photography (FP), and Fluorescein Angiography (FA) that provides micro-vascular insights cheaper and easier than MRI systems^5,6^.

OCTA Scan produces images of the complex network of retinal micro-blood vessels. Brain Structure presents the direct neurovascular connection of the retina to the Central Nervous System. The optic nerve functions as Optca, enabling neural signals to bridge between retina cells and brain tissue, revealing the existence of vascular-nerve connectivity. Neural signals generated by retinal ganglion cells travel through the Optic Nerve Conduit (Optca) to reach the brain while reflecting the condition of cerebral micro-vessels. The conduit functions as a pivotal diagnostic tool because retinal micro-vascular issues tend to occur before or show identical patterns to white matter hyperintensities of CSVD. White matter hyperintensities appear as a fundamental diagnostic marker of CSVD, which shows micro-vascular ischemic changes and demyelinating effects^7^. This image demonstrates the importance of retinal biomarkers, which include Foveal Avascular Zone (FAZ) enlargement and measurements of caliber size, as well as the assessment of tortuosity and evaluation of capillary loss. The expansion of the FAZ area shows that ischemic conditions exist within the foveal portion of the retina. An indicator of deficient micro-vascular blood flow is capillary loss. Tortuosity shows vascular instability along with abnormal self-regulatory problems. The condition of retinal vessel diameter either becoming narrower or wider indicates cerebral vascular complications. Researchers have identified biomarkers that strongly link to cerebral micro-angiopathic changes because they can predict CSVD advancement at early stages^8^.

CSVD can be well detected through retinal biomarker analysis due to similarities in microvasculature in the eye and the brain. Retinal imaging technologies (FP, OCTA and FA) have been used to find changes in small vessels detected early as indicators of ischemia and neurodegeneration that can be associated with CSVD. Key biomarkers are retinal nerve fibre layer thinning, enlargement of the foveal avascular zone, vessel tortuosity, capillary dropout and a decreased Arterio-Venous Ratio (AVR). These features correlate highly with volumes of White Matter Hyperintensities (WHM), a well-established marker of CSVD, on MRI scan. Thus, retinal abnormalities reflect the Cerebro-cortical micro-angiopathic processes, and thus provide a non-invasive, low cost and scalable mechanism for screening CSVD early. In this study, a framework is presented to extract and correlate these retinal biomarkers using deep learning utilizing cerebral changes. Thus, enabling the system to predict and detect CSVD before symptoms become evident.

The current CSVD screening methods do not have a unified, scalable system that combines multimodal retinal biomarkers for accurate risk prediction of CSVD, though retinal imaging and computational diagnostics have made significant improvements. The currently implemented frameworks either work with a single imaging modality, fail to utilize retinal vascular network connectivity patterns or do not have cross-dataset generality capabilities. The current framework lacks the means to unite deep spatial-temporal learning approaches with functional retinal correlation structures while remaining adaptable to treat diverse clinical conditions.

As a solution to current framework deficiencies, the Retino-Neuro Vision Transformer (RNV-T) framework serves as a new five-phase deep learning system developed to detect the initial stages of CSVD through multimodal retinal image examination. An innovative pipeline for early CSVD detection uses five sequential components consisting of Local Vascular Extraction (LVE) through retinal feature decomposition and Global Transformer-based Encoding (GTE) for spatial context integration followed by Graph-based Convolutional Attention Network (G-CAN) to extract topological connections and Local-Global Attention Fusion (LGAF) for hierarchical insight combination as well as Federated Meta-Optimized Training for model adaptability and data privacy protection. RNV-T establishes an end-to-end extraction system that detects both local lesion-specific biomarkers and patient-level spatial linkages in CSVD data, unlike previous detection methods.

### Novelty and significance

RNV-T demonstrates its unique value through the combination of graph theory with transformers and federated learning techniques used for retinal vasculature assessment toward cerebrovascular risk assessment. The approach differs from earlier methods by integrating information about retinal context and connection patterns and spatial progress through topological embedding techniques and attention mechanisms, which demonstrate combined structural (Retinal Nerve Fiber Layer-RNFL) thinning conditions with perfusion (FAZ enlargement, leakage zones) abnormalities^9^. The model architecture gives rise to an easy-to-understand diagnostic solution that displays superior diagnostic capability beyond standard classification methods.

### Scope and motivation

The research sets opportunities for the use of cost-effective ophthalmic imaging systems as part of neuro-diagnostic processes, especially where limited resources exist. The foundational purpose exists to perform early noninvasive CSVD risk assessment while decreasing MRI dependency and supporting large-scale retinal device screening to enhance diagnostic equality.

### contributions

The main contribution of this research involves creating the RNV-T framework that presents an innovative method to detect early signs of CSVD through non-intrusive and affordable multimodal retinal imaging examinations. RNV-T establishes a new standard in micro-vascular abnormality detection because of its transformer-based global encodings and graph-convolutional relational learning together with federated meta-optimization capability, which delivers optimal accuracy. The technical application of this framework enables real-time interpretive assessment of risk levels through accessible ophthalmic tools and guarantees scalable operation, which makes it independent of expensive MRI testing. This technological advancement provides communities with screening capabilities at local levels in areas with limited healthcare infrastructure; thus, it enables prompt neurological intervention while improving access to equitable and inexpensive eye-based diagnostic services that decrease cognitive deterioration combined with stroke risks.

### Research objectives

The core tasks of this research study are as follows:

- To produce the RNV-T architecture, which unites transformer methods and graph-based analytics for CSVD detection from combination retinal data.
- To evaluate RNV-T diagnostic capability against established models by applying real-world information from retinal FP, OCTA, and FA modalities.
- Graph-level feature interpretation and attention mechanisms are employed to identify critical retinal biomarkers associated with WMH and CSVD.

The research manuscript uses six fundamental sections to organize a complete technical presentation of the study. Section  1 includes the introduction that describes the background of CSVD alongside its retinal imaging motivation and existing limitations that led to the development of the proposed RNV-T framework. Section  2 examines past studies dedicated to retinal-based cerebrovascular assessment while pointing out technical shortcomings alongside available opportunities. The detection methodology of RNV-T progresses through five stages, from Local Vascular Extraction to Federated Meta-Learning Optimization, as explained in Sect.  3. In Sect.  4, materials utilized describe the multiple retinal datasets (FP, OCTA, FA) together with the software hardware components that were used. Section  5 presents the diagnostic performance evaluation analyses of the RNV-T Framework by assessing accuracy, sensitivity, and specificity measurements, as well as benchmark comparisons. The study ends with Sect.  6, which presents the summary of the study, key research outcomes, and societal impacts while setting future research agendas.

## Literature work

Research in^10^ demonstrated that retinal vessels share anatomical and physiological similarities with cerebral vessels while advocating retinal digital analysis as a suitable method for both non-invasive and economical screening of cerebrovascular diseases with an emphasis on examining small-vessel pathologies. The research used comparative anatomical examinations together with literature reviews about vascular imaging approaches and epidemiological database analysis to determine the relationship between retinal biomarkers and cerebral disease risk factors, particularly stroke and dementia. A combination of vessel diameter assessments together with bifurcation analysis and auto-regulatory mechanism evaluation yielded significant results, which demonstrated that people with focal retinal abnormalities carried a 3.0 relative risk (corresponding to a 300% greater stroke risk). Retinal imaging serves as a useful brain assessment alternative but has not yet reached the spatial definition or depth of Magnetic Resonance Imaging (MRI) for cerebral evaluation.

The DL model developed by^11^ uses 7-layer convolutional neural networks (CNN) to evaluate preprocessed MRI segmentations into 7 × 7 patches for identifying CSVD biomarkers. The implemented system delivered better results than Multi-layer Perceptron (MLP), and You Only Look Once (YOLO) variants through its 98.57% training accuracy and 98.52% verification accuracy. The in-depth lesion images provided by MRI create challenges for routine screening due to its length and high resource requirements; thus, image-based testing becomes more suitable for widespread applications.

The study in^12^ conducted a thorough evaluation of ML predictive models in incident dementia forecasting for CSVD patients across three major international research groups through their baseline measurement datasets. This research conducted survival analysis along with classification analysis to predict 3-year risks. It tested regularized cox/logistic regression, Support Vector Machine (SVM), Random Survival Forests (RSF), Gradient Boosted Trees (GBT), Generalized Matrix Learning Vector Quantization (GMLVQ), and Generalized Relevance Learning Vector Quantization (GRLVQ) models using MRI-based CSVD markers together with cognitive scores and demographic characteristics. Regularized regression models proved to be superior to least demanding ML approaches according to core results because these models generated the best C-index (0.863) and Area Under the Curve (AUC) (0.870) metrics even though ML showed no statistically significant gains over regularized regression models. Global cognition paired with chronological age showed themselves to be the strongest predictors throughout the analysis, although brain volume data contribution proved limited. The study encountered limitations from MRI features’ limited predictive capability since cognitive assessments demonstrated better predictive strength, although they pose a large clinical burden.

Author in^13^ designed a dual-task DL framework by combining Mask R-CNN with Multi-Instance Learning for analysis of susceptibility-weighted MR sequences (SWS) to identify cerebral micro-bleeds (CMBs) in addition to distinguishing cerebral small vessel disease (CSVD) categories as Arteriolosclerosis (aSVD) and Cerebral Amyloid Angiopathy (CAA) and Cerebral Autosomal Dominant Arteriopathy with Subcortical Infarcts and Leukoencephalopathy (CADASIL). The detection model performed at a 70.6% recall rate for CMB findings while attaining 90.8% area under the curve for CSVD category assessment, thus exceeding results from neurologist standards. The widespread adoption of simpler DL methods appeals as a solution to the complexity of MRI diagnosis because it requires multiple sequences.

The work in^14^ provided an extensive review of DL methods that analyze CSVD features through MRI scans by examining important indicators, including lacunes alongside brain atrophy. The research methodology used CNNs and segmenting structures (U-Net) and detected objects (Mask Region-CNN and YOLO), which demonstrated segmentation performance through Dice scores up to 84.01% and detected object progressions through F1 scores up to 74.74%. The main drawback of multimodal MRI technology comes from its complex nature, which renders it difficult to scale for clinical operations because retinal image-based screening proves more effective.

Investigators developed a Sparse Bayesian Feature Selection (SBFS) model that integrated retinal fundus and OCT imaging results together with demographic information to determine CSVD severity levels^15^. The provided model demonstrated 71.71% accuracy and 73.35% AUC by showing global venous width along with other retinal biomarkers as fundamental predictors. Research findings validate the usefulness of ophthalmic imaging as a method to screen CSVD since this approach costs less and is simpler to access than MRI technology, but MRI still poses restrictions because of the expense involved and the infrastructure requirements.

Recent study implemented EVision Artificial Intelligence (AI) analysis with MRI to assess retinal vein occlusion types Branch Retrovascular Occlusion (BRVO) and Central Retrovascular Occlusion (CRVO) by analyzing retinal and cerebral vascular microstructures^16^. The research used ResNet101-U-Net and Single Shot MultiBox Detector (SSD) to segment retinal images, which demonstrated wider optic nerve subarachnoid space width (ONSASW) and enlarged venular calibers were important markers for CRVO, but BRVO revealed lower Ophthalmic Artery (OA) diameters and cerebrovascular traits. Understanding retinal imaging as a substitute for practical population-wide screening remains possible because MRI presents both high-cost barriers and operational limitations.

Researchers in^17^ applied VB-Net to multi-modality MRI to identify and measure the five CSVD markers: WMH, Recent Small Subcortical Infarcts (RSSI), Lacunar Infarcts (LI), Perivascular Spaces (PVS), and Cerebral Microbleeds (CM). This system reached segmentation Dice scores higher than 0.90 and recall/precision levels up to 97.2%/93.3% (for PVS). The combination of clinical risk factor analysis through regression analysis confirmed age and SBP together with total CSVD score as stand-alone predictors for lacunar stroke. The high-accuracy performance of MRI is limited for clinical scalability because multiple sequences require expert validation during the process.

Research contributors in^18^ presented the Binocular Fundus Image – Age-Related White Matter Changes (BFI-ARWMC) framework that involves binocular retinal fundus images to detect White Matter Hyperintensities (WMH) in ischemic stroke patients through MRI-derived ARWMC scoring reference standards. The implementation using ResNet50 together with Global Average Pooling detected WMH in stroke patients with 81.1% Macro Accuracy and F1 score affirmation for non-invasive assessment. A main drawback arises from the use of MRI as a gold standard method since it remains too expensive and impractical for large-scale population screening, which makes retinal imaging an attractive alternative. Table 1 represents the summarized overview of the prominent existing works.

**Table 1:** Summarized highlights of existing studies with key points on the investigation **of CSVD**.

Author	Method	Focused on	Attainments (%)	Remarks
^10^	Retinal Image Analysis	Cerebrovascular disease screening	Up to 300% increased stroke risk (RR = 3.0)	Retinal imaging lacks spatial precision
^11^	Patch-CNN (7-layer)	MRI-based CSVD lesion detection	98.57% (train), 98.52% (val)	Limited for rapid screening
^12^	SVM, RSF, GBT, GMLVQ and GRLVQ	Dementia risk in SVD	ROC-AUC up to 87.0%	MRI biomarkers less predictive than cognition
^13^	Mask R-CNN + MIL	CMB detection + CSVD classification	Recall: 70.6%, AUC: 90.8%	MRI complex; multimodal needs hinder practical use
^14^	CNN, U-Net, Mask R-CNN	MRI-based CSVD lesion analysis	Dice = 84.01%, F1 = 74.74%	Complexity and resource-intensiveness
^15^	SBFS	Retinal-based CSVD detection	ACC = 71.71%, AUC = 73.35%	Retinal imaging enables scalability
^16^	AI-based MRI (EVision)	Retinal vs. cerebral vessel traits in CRVO vs. BRVO	Statistically significant biomarkers (*p* < 0.05)	Limits mass screening
^17^	VB-Net (DL)	MRI-based CSVD quantification	DSC > 0.90; PVS Recall = 97.2%	MRI requires multi-sequence, expert correction
^18^	BFI-ARWMC (DL)	WMH prediction via retina	F1 = 81.1%, ACC = 81.1%	MRI expensive, limited accessibility in screening

The reviewed studies demonstrate the use of MRI and retinal imaging techniques for detecting CSVD through methods that include CNNs and patch-based segmentation, Bayesian feature selection, and advanced ensemble DL architectures. Research using MRI technologies demonstrated excellent accuracy and precise lesion detection yet dealt with three significant challenges regarding cost expense and restricted availability together with complex computational needs. Fundus and OCT retinal scans proved effective in the moderate detection of diseases since they revealed structural information about retinal vascular networks, including global venous width and tortuosity that strongly reflect brain micro-vascular conditions. The identification of retinal imaging as an essential tool for CSVD diagnosis emerges from research that establishes it’s low-cost and scalable modality along with non-invasive features that make it an alternative to MRI specifically suited for mass screening at eye clinics, especially for underserved nations.

## Detection methodology

**Fig. 1:**
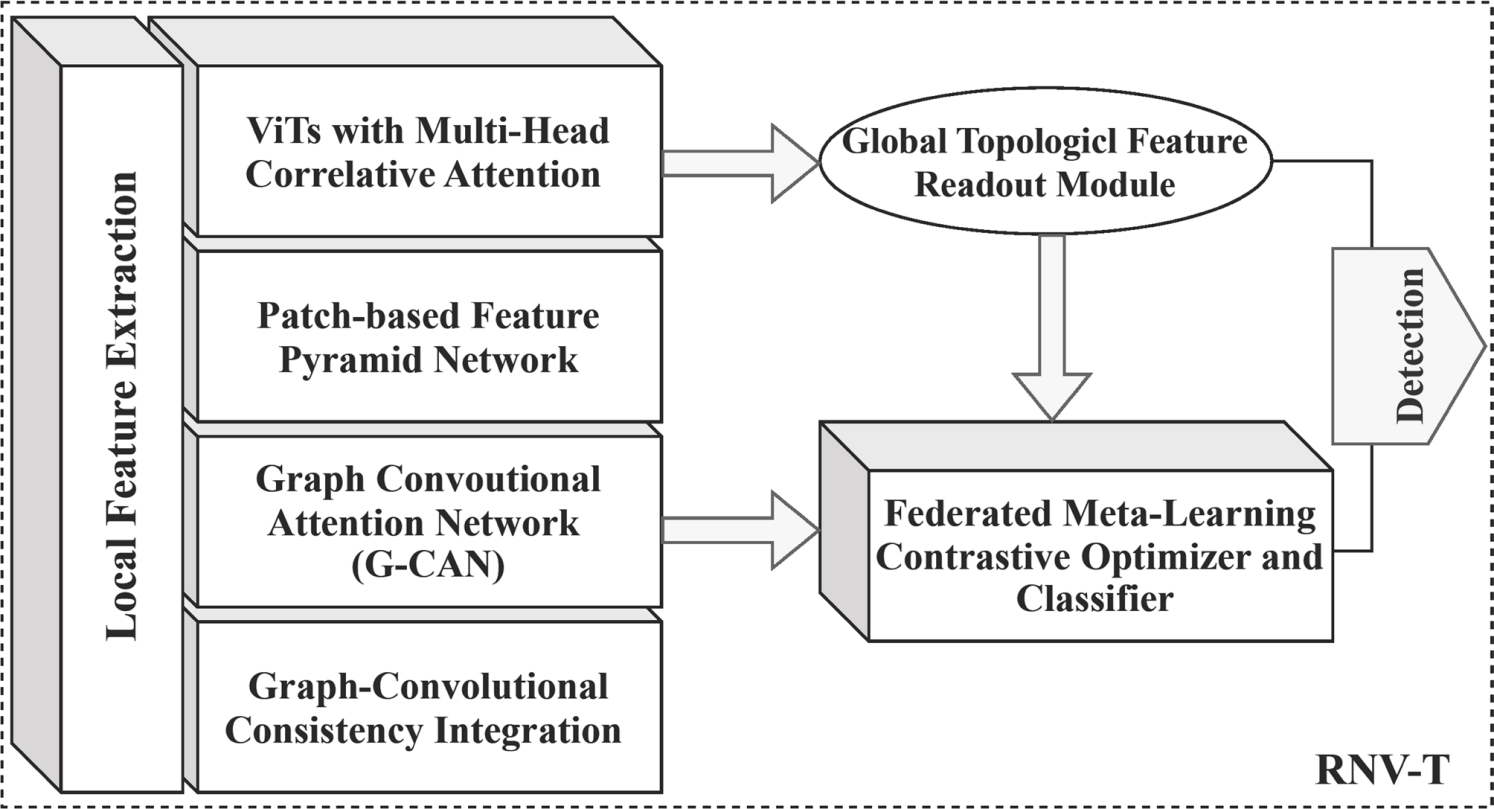
Architectural overview of RNV-T.

The research introduces a novel DL-based framework to detect CSVD at its early phases through the evaluation of multiple retinal imaging modalities. Figure 1 illustrates the RNV-T framework architecture that consists of five distinct phases for operation. The phases of the framework are LVE, GTE, G-CAN, LGAF, and Optimized Training. The phases operate together to increase representation capabilities between morphological local features, global spatial knowledge, and topological structure analysis.

The RNV-Transformer uses a multi-stream architecture that combines CNN extraction of localized vascular features with vision transformer wide-field pattern recognition and G-CAN for topological relationship learning. The system performs highly accurate biomarker prediction for CSVD directly from retinal scans by using LGAF together with adaptive self-supervised pre-training and federated meta-learning.

### Phase 1: local vascular extraction

The initial component of RNV-Transformer uses CNNs to extract complex morphological features that exist in retinal vasculature patterns. High-resolution fundus photography (*FP*) data, together with OCTA and fluorescein angiography (*FA*), yields crucial patterns, including vessel narrowing, hemorrhages, tortuosity, and micro-aneurysms during this processing stage. Let the input of retinal imaging modalities (*m*) be $\:{\:\mathbb{I}}_{\left(FP\left|\left|OCTA\right|\right|FA\right)}\in\:{\mathbb{R}}^{\left(height\times\:width\times\:{channel}_{i}\right)}$. The CNN architecture progresses through spatial levels to decipher edge features and split patterns as well as small vessel outlines using significant operational components like convolution (*), Rectified Linear Unit (ReLU) activation function (*λ*), and future mapping $\:{f}_{0}^{\left(m\right)}$. Thus, the modality-based convolutional encoding for future mapping is defined as,

1 $$\:{f}_{l}^{\left(m\in\:FP,OCTA,\:FA\right)}=\lambda\:\left[{f}_{l}^{\left(m\right)}*{\omega\:}_{l}^{\left(m\right)}+{e}_{l}^{\left(m\right)}\right],\:l=\mathrm{1,2},\cdots\:,{L}_{m}\:and\:{f}_{0}^{\left(m\right)}\leftarrow\:{\mathbb{I}}^{m}$$

In (1), $\:{\omega\:}_{l}^{\left(m\right)}$denotes the kernel weights (convolution) specific to layer *l* and modality *m*, $\:{e}_{l}^{\left(m\right)}$ refers to the error correction or bias term signal at *l*^*th*^ layer, and $\:{f}_{0}^{\left(m\right)}\leftarrow\:{\mathbb{I}}^{m}$ establishes that the initial feature map is the input image of *m*. The design relies on multi scale pooling functions to identify different scale vessels in order to ensure that the vascular structures of all thickness and resolution are thoroughly examined. Thus, such multi-resolution vascular data is extracted as pyramidal future mapping and expressed as,

2 $$\:{F}_{pyr}^{\left(m\right)}={\oplus\:}_{s=1}^{S}{R}_{s}\cdot \:\left({f}_{{L}_{m}}^{\left(m\right)}\right)$$

In Eq. (2), $\:{R}_{s}$ signifies the effect of spatial rescaling to scale *s* using depth-wise concatenation (⊕). This computation highly preserves the hierarchical patterns across different vessels and modalities. The processed features are transformed through a multi-layer perceptron (MLP)^19^, which generates organization of embedded representations to represent vascular biomarkers. These embeddings contain a lot of vessel caliber measurements, AVR data, and branching angular information, which are not directly measured at this stage, but still keep discriminative structural information, which is extracted from the retinal vasculature. Consequently, MLP processed embeddings are able to capture morphological cues such as vessel caliber and AVR, belonging to branched and complex vessels, that correlates with vascular biomarkers, and is utilized at later stages of transformer and graph-based learning. Thus, each pyramid aggregated features are transformed into vector forms and expressed as,

3 $$\:{\overrightarrow{v}}^{\left(m\right)}={MLP}^{\left(m\right)}\cdot \:\left(flattent\left({F}_{pyr}^{\left(m\right)}\right)\right),\:where\:{\overrightarrow{v}}^{\left(m\right)}\in\:{\mathbb{R}}^{d}$$

In Eq. (3), $\:{\overrightarrow{v}}^{\left(m\right)}$ captures the modality-based vascular patterns. Further, on utilizing the Hadamard product (⊙), a non-linear cross-modal transformation computation (for cross-correlated fusion) is handled as^20^,

4 $$\:{\overrightarrow{v}}_{fusion}^{\left(m\right)}={\zeta\:}_{corr}\left({\overrightarrow{v}}^{FP},{\overrightarrow{v}}^{OCTA},{\overrightarrow{v}}^{FA}\:\right)\to\:{MLP}_{f}\left({\overrightarrow{v}}^{FP}\odot\:{\overrightarrow{v}}^{OCTA}+{\overrightarrow{v}}^{FA}\right)$$

In (4), ⊙ captures the inter-modality correlation. Finally, the local feature representation in this phase receives combined features from all modalities through a refined vascular descriptor vector to produce essential localized information for global relation analysis, which is represented as,

5 $$\:{\stackrel{\sim}{f}}_{cnn}={Norm}_{L}\left({MLP}_{refined}\left({\overrightarrow{v}}_{fusion}^{\left(m\right)}\right)+{\overrightarrow{v}}_{fusion}^{\left(m\right)}\right)$$

The fused vector $\:{\stackrel{\sim}{f}}_{cnn}$ from Eq. (5) acts as the multi-model feature representer for subsequent/incidental transformer processing. Altogether, the entire procedure of local vascular extraction in phase 1 is depicted in an algorithmic form in Table 2.

**Table 2:**
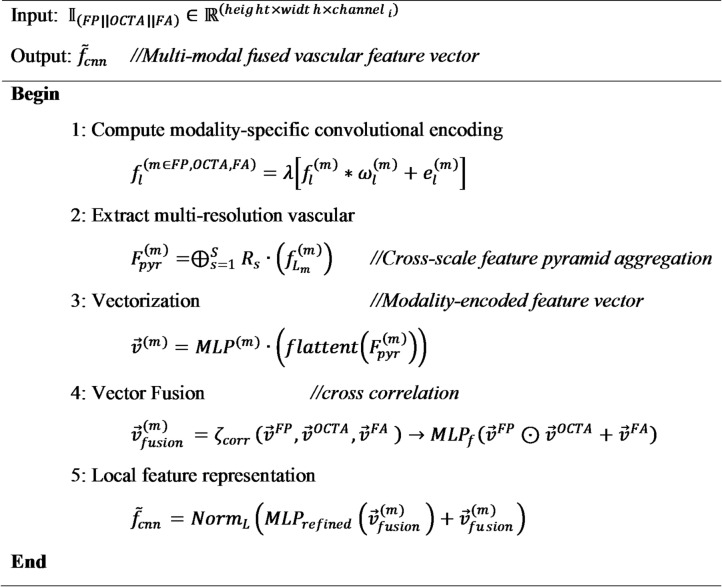
Algorithmic procedures of phase 1.

### Phase 2: global transformer-based encoding

The model progresses from attending to regional retinal features during phase 1 to using a Vision Transformer (ViT) backbone that analyzes overall retinal vascular maps and spatial arrangement patterns during phase 2^21^. The ViT module detects structural elements found within the anatomical structure, including FAZ enlargement and capillary loss. A fixed-size partitioning of the image results in patches $\:\left({\rho\:}_{i}\in\:{\mathbb{R}}^{\left(\rho\:\times\:\rho\:\times\:{channel}_{i}\right)}\right)$ that lead to linear embedding generation (extract token embeddings) before adding specialized positional encoding to preserve topological awareness. Thus, patch tokenization (Eq. 6) and position embeddings (Eq. 6) are computed as follows:

6 $$\:{T}_{i}=\left(flatten\left({\rho\:}_{i}\right)\cdot \:{\omega\:}_{\rho\:}\right)+{e}_{\rho\:}$$

In positional encoding (*ψ*) for spatial sensitivity, quadratic sinusoidal encoding is employed.

7 $$\:{\psi\:}_{i}^{\left(d\right)}=\mathrm{sin}\left[\raisebox{1ex}{${\pi\:\cdot \:i}^{2}$}\!\left/\:\!\raisebox{-1ex}{${10000}^{\left(d/D\right)}$}\right.\right]$$

From (7), *d* specifies the specific dimension index across the overall dimensionality of the embedding space (*D*) and *π* is the domain-stated encoding operation. Beyond conventional transformers, this phase employs a Multi-Head Correlative Attention (MHCA) process (*Φ*) that measures both key-query pair similarity and learns to encode divergence through trainable correlation terms. The model operates through two parallel mechanisms to identify both minor vascular pattern variations together with major FAZ size abnormalities and perfusion empty areas. The computational data points from token interactions reveal the co-association patterns and divergent characteristics between separate parts of the retinal vascular network.

8 $$\:\varPhi\:\left({\tau\:}_{q},{\tau\:}_{k},{\tau\:}_{V}\right)={\sum\:}_{h=1}^{{H}_{i}}{Z}_{h}\cdot \:softmax\left[\raisebox{1ex}{$\left({\tau\:}_{q}\cdot \:{\tau\:}_{k}^{\left(\epsilon\:\right)}\right)+l{\left({\tau\:}_{q}\cdot \:{\tau\:}_{k}\right)}^{2}$}\!\left/\:\!\raisebox{-1ex}{$\sqrt{{d}_{k}}$}\right.\right]{\tau\:}_{V}$$

From (8), $\:{\tau\:}_{q},{\tau\:}_{k},{\tau\:}_{V}$ denotes the tokenizing components query retinal patch, key retinal patch, and value retinal patch embeddings, respectively. Further *h*, *l*,* ε* and Z denotes the head index, cross-token learnable correlated modulation component, token embedding sequences and learnable parameters, respectively. Through its ability to combine the representations of encoded tokens, the transformer generates a global representation of retinal vascular maps, and it reveals inconspicuous biomarkers that point to systemic cerebrovascular disease. Thus, the updated token embedding after employing *Φ* and normalization process is computed as,

9 $$\:{\epsilon\:}^{{\prime\:}}={Norm}_{L}\left(\varPhi\:\left({\tau\:}_{q},{\tau\:}_{k},{\tau\:}_{V}\right)\right)$$

The resulting global feature vector (in Eq. 10) emerges from the transformer encoding of the classification token (CT) token to consolidate spatial data across the entire retinal network.

10 $$\:{\overrightarrow{\mathcal{G}}}_{retina}=MLP\left[CT\left({\epsilon\:}^{{\prime\:}}\right)\right]$$

Table 3 represents the algorithmic processes of phase 2 (global transformer-based encoding).

**Table 3:**
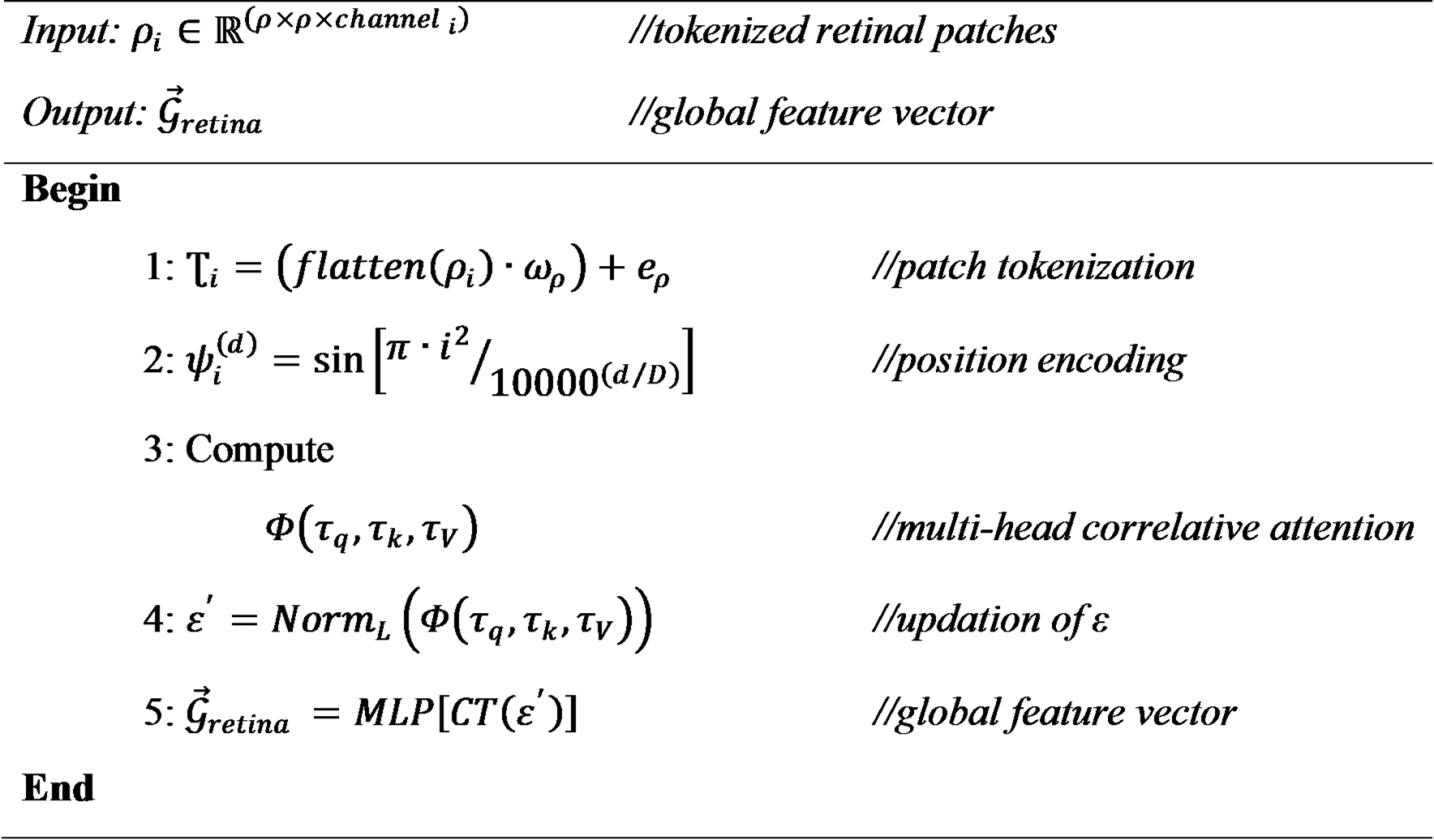
Algorithmic processes of phase 2.

### Phase 3: graph-based retinal relational learning

Phase 3 performs topological and relational evaluation through G-CAN by establishing a graph structure $\:\mathbb{G}=\left\{J,C\right\}$ that encompasses retinal vascular networks. Each structural unit within this graph stands for vascular endpoints or junctions (*J*_*i*_), while vessel segments serve as connections (*C*_*j*_) between them. The relational model detects vessel connections together with branching angles as well as complex topological features which are not processed independently through grid-based approaches. An adjacency matrix data $\:\left({\ddot{a}}_{ij}\right)$ represents the construction through proximity analysis along with continuous vessel attributes, which are weighted by exponential force to reinforce main connections and such computation is defined as,

11 $$\:{\ddot{a}}_{ij}=exp\left(\frac{1}{{\|{\delta\:}_{i}-{\delta\:}_{j}\|}^{2}/s}\right)\cdot \:{1}_{connected}\left(i,j\right)$$

From (11), $\:\delta\:$ and *s* defines the spatial coordinates in the retinal vascular networks and scaling factor, respectively. Further, the approach applies graph-convolutional attention-based message-passing mechanisms to discover important relationships within vessel segments by adjusting weights for their local neighborhood understanding, which is computed as,

12 $$\:{h}_{i}^{(l+1)}={\uplambda\:}\left[{\sum\:}_{j\in\:\mathbb{N}\left(i\right)}\left({\omega\:}_{ij}^{\ddot{a}}\cdot \:\left|L\right|{h}_{i}^{\left(l\right)}\right)\right]$$

From (12), at each layer, the node feature vector is computed via vital components like attention weight between *i* and *j* $\:\left({\omega\:}_{ij}^{\ddot{a}}\right)$, learnable transformation matrix $\:\left|L\right|$, and neighbor node of *i*, $\:\mathbb{N}\left(i\right)$. Then the system incorporates an innovative anomaly detection technique that measures the difference between real-world (actual) and predicted attention weight data to identify irregular micro-vascular connections that standard transformers overlook, which is referred as in (13).

13 $$\:{\ddot{a}}_{ij}=\left|{\omega\:}_{ij}^{\ddot{a}}\left(actual\right)-{\omega\:}_{ij}^{\ddot{a}}\left(expected\right)\right|$$

The graph-level representation combines last-layer node features while including both local vascular relationships and their measured topological anomaly scores, which is expressed as,

14 $$\:{\overrightarrow{g}}_{\mathbb{G}}=readout\left[{\left({h}_{i}^{\left(L\right)}\right)}_{i=1}^{\left|J\right|}\right]$$

The representation in (14) measures multiple hierarchies of relationships across several layers that indicate disruptions in the micro-vasculature characteristic of CSVD. Finally, the relational patterns between retinal vessels within retinal images produce an aggregated graph-level representation that generates a feature vector $\:\left({\overrightarrow{v}}_{relational}\right)$ that represents cerebrovascular health through network behavior.

15 $$\:\left({\overrightarrow{j}}_{relational}\right)=MLP\left({\overrightarrow{g}}_{\mathbb{G}}\right)$$

Table 4 represents the algorithmic processes of phase 3 (graph-based retinal relational learning).

**Table 4:**
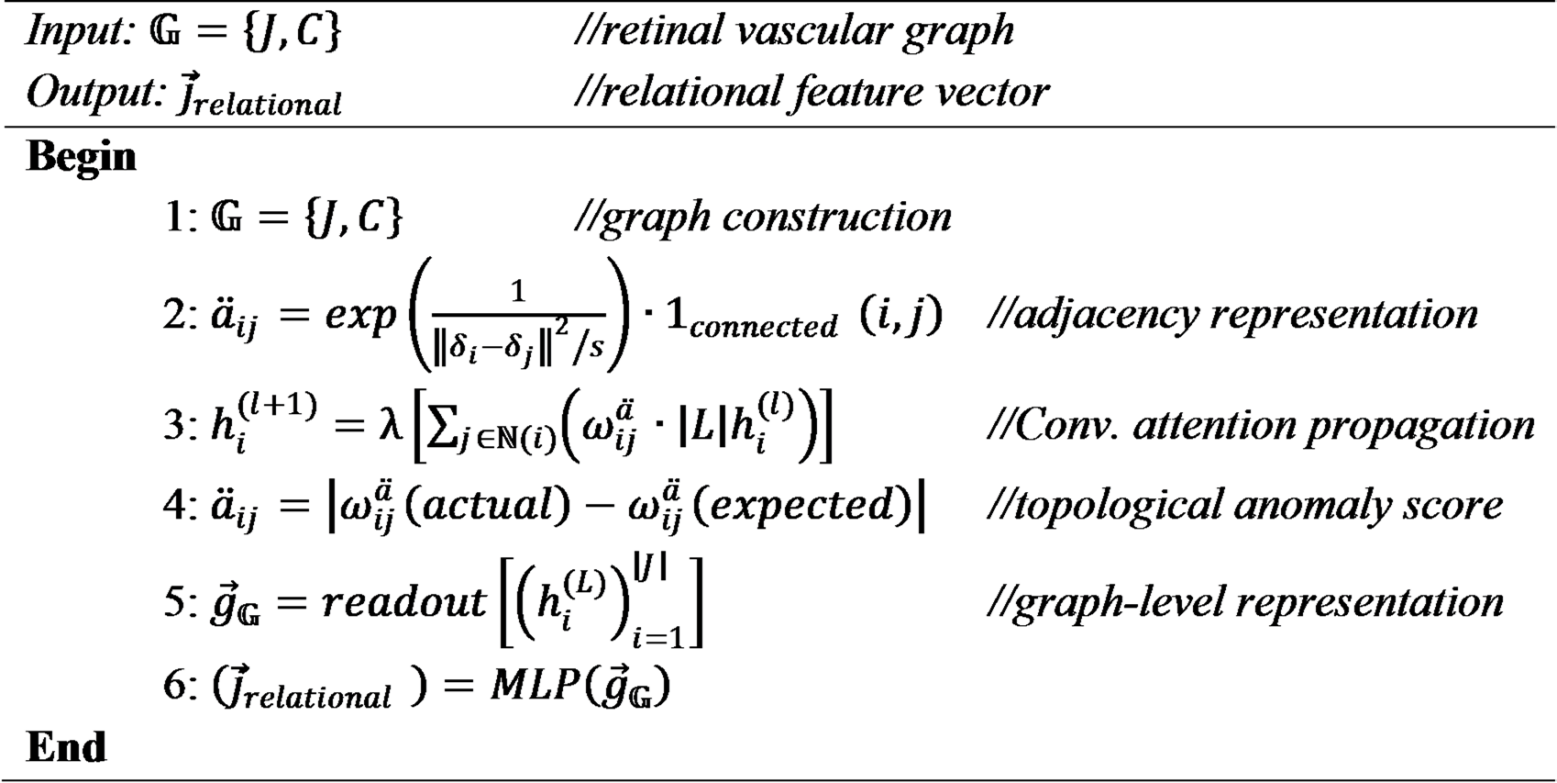
Algorithmic procedures of G-CAN.

### Phase 4: local-global attention fusion

The LGAF module performs the fourth stage by uniting independently derived local and global vascular features. The LGAF module (*η*) combines intermediate features $\:\left({\overrightarrow{\phi\:}}_{i}\right)$ by weighting transforms between localized CNN vessel attributes along with globally contextualized transformer output. For this, an adaptive gating system $\:\left({\varTheta\:}_{i}\right)$ acquires significant weight estimates according to contextual features, meaning that the model chooses vital information sources based on treatment conditions, which is defined as in Eqs. (16) and (17).

16 $$\:{\overrightarrow{\phi\:}}_{i}=\left[\left({\overrightarrow{v}}_{fusion}^{\left(m\right)}{\cdot \:\varTheta\:}_{i}\right)+{\overrightarrow{\mathcal{G}}}_{retina}\cdot \:\left(1-{\varTheta\:}_{i}\right)\right]$$

17 $$\:{\varTheta\:}_{i}=\lambda\:\left[{\omega\:}_{\mathcal{G}}\cdot\:\left\{{\overrightarrow{v}}_{fusion}^{\left(m\right)}\left|\right|{\overrightarrow{\mathcal{G}}}_{retina}\right\}+{e}_{\mathcal{G}}\right]$$

The approach applies an attention-based aggregation process to learned fusion vectors that select significant diagnostic information within the retina regions, which is computed as,

18 $$\:{\overrightarrow{f}}_{\eta\:}={\sum\:}_{i}softmax\left({{\rm\:Y}}_{i}\right)\cdot \:{\overrightarrow{\phi\:}}_{i}$$

From (18), $\:{{\rm\:Y}}_{i}$ indicates the attention coefficient between G-CAN nodes, which determines the importance of the weight of fused vectors to improve discriminative features for CSVD classification, especially during aggregation process. Further, a consistency-aware modulation term enables graph-derived relational features to be added into the fused representation to maintain consistent information across different representation types, which can be represented as,

20 $$\:{\overrightarrow{f}}_{O}={\overrightarrow{f}}_{\eta\:}+S\cdot \:{\overrightarrow{j}}_{relational}$$

In (20), the scaling factor *S* balances between relational G-CAN features and the local-global representation in the fusion mechanism. Thus, the final predicted outcome is computed as,

21 $$\:{\widehat{Y}}_{csdv}=softmax\left({\overrightarrow{f}}_{O}\cdot \:{\omega\:}_{O}+{e}_{O}\right)$$

Thus, the fusion strategy enables the model to maintain detailed information about vascular networks while understanding structural patterns across the retina. It also captures relational vascular relationships, so it produces an optimized feature vector for detecting CSVD. The entire computational processes of LGAF in phase 4 is depicted as algorithmic procedure in Table 5.

**Table 5:**
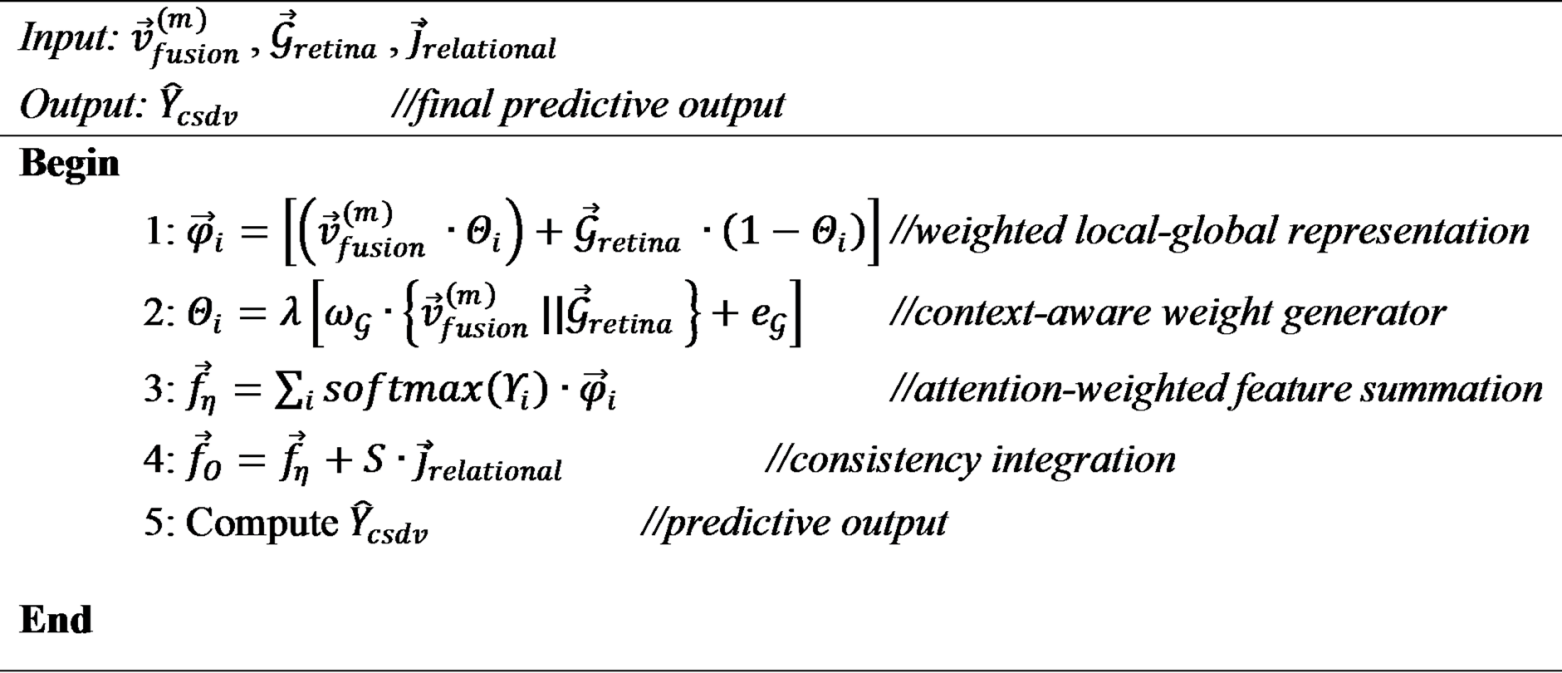
Computational processes of LGAF in phase 4.

### Phase 5: optimized training

The last stage (phase 5) uses adaptive self-supervised learning (SSL)^22^ alongside federated meta-learning (FML)^23^ to optimize the RNV-T’s learning process. The self-supervised phase of the model trains robust representations by optimizing agreement between different retinal sample views, which undergo augmentation without specific labels. Thus, the contrastive objective of SSL (per modality) is computationally represented as,

22 $$\:{\mathbb{L}}_{ssl}=\left\{1/\mathrm{l}\mathrm{o}\mathrm{g}\left(\frac{exp\left(\nabla\:\langle{d}_{i},{d}_{j}\rangle/{t}_{p}\right)}{{\sum\:}_{k=1}^{N}exp\left(\nabla\:\langle{d}_{i},{d}_{k}\rangle/{t}_{p}\right)}\right)\right\}$$

Thus, the contrastive learning approach enables the network to better understand small pattern variations occurring in human retinas among different patients. The integration of FML in proposed RVN-T enables multiple healthcare centers to conduct training activities independently without distributing original retinal data. The model uses this security technology because it ensures population diversity in data analysis without violating individual patient data privacy, which remains essential in the AI-based medical domain. During the research, this study enables the RNV-Transformer to receive localized training through FML at diverse hospitals, which extracts institution-specific CSVD patterns. Central servers use model parameters from different clinics to aggregate and adapt them for generalized learning in order to produce robust privacy-aware CSVD predictions between various clinical sources. The core computation of FML is defined as,

23 $$\:\underset{\vartheta\:}{\mathrm{min}}{\sum\:}_{k=1}^{K}{\mathbb{L}}_{{T}_{k}}\cdot \:\left[{\mu\:}_{k}\left(\vartheta\:\right)\right]$$

The computation exhibited in (23) reflects the task-specific loss $\:\left({\mathbb{L}}_{{T}_{k}}\right)$ for the any *k*^th^ client in FML, while $\:{\mu\:}_{k}\left(\vartheta\:\right)$ denotes the update retrieved from the local data repository at *k* and $\:\vartheta\:$ signifies the models parameters shared among all clients and server. Further, the approach combines three different objectives into its final loss function (Eq. 24) to achieve accurate supervised classification (*asc*) while performing self-supervised consistency tests and maintaining relational graph regularization.

24 $$\:{\mathbb{L}}_{overall}={\sigma\:}_{1}{\mathbb{L}}_{asc}+{\sigma\:}_{2}{\mathbb{L}}_{ssl}+{\sigma\:}_{3}{\sum\:}_{{\overrightarrow{j}}_{relational}}{\ddot{a}}_{ij}$$

From (24), each weight component $\:{\sigma\:}_{1},{\sigma\:}_{2},{\sigma\:}_{3}$ helps balance the strength of individual losses included in the overall objective. In addition, the final optimization step represented in Eq. (25) includes a modified Adam optimization algorithm to perform learning update adjustments based on gradient magnitude calculation and consistency constraint enforcement.

25 $$\:{\xi\:}_{t+1}={\xi\:}_{t}-\alpha\:\cdot \:\left[\frac{{A}_{t}}{\left(\sqrt{{B}_{t}}\right)+{\hat{s}}+{\|\nabla\:{\mathbb{L}}_{overall}\|}^{2}\cdot \:{\check{g}}}\right]$$

In (25), the model receives parameters *ξ* at training step *t* followed by parameters at step *t + 1*. During training, the learning rate *α* acts as the modifier, although it updates the model parameters. Moreover, the first-order moment (approximation) $\:{A}_{t}$ of gradients serves as the momentum terms in adaptive optimizers, which eventually leads to the inclusion of second order variance$\:\sqrt{{B}_{t}}$. Further, an additional small value *ŝ* added to the denominator serves as a numerical stabilizer during optimization. For optimization purposes, the end process uses the total loss gradient $\:\left(\nabla\:{\mathbb{L}}_{overall}\right)$ as a derivative of model parameters along with the scaling factor $\check{g}$ for gradient-based consistency acts as an updater that controls gradient-based updates through gradient norm observations. As a result, these analytical techniques allow the RNV-T to sustain accurate diagnoses across diverse clinical data conditions.

## Materials utilized

### Experimental components

Open-source software frameworks provided the basis for experimental research through their development of all implemented components. Developing the major DL models relied on Python v3.9 as the programming language, while PyTorch v1.13.1 functioned for model building and training alongside TorchVision v0.14 for image processing. Retinal image enhancement and morphological operations were executed with OpenCV v4.5.5 and scikit-image v0.19.3 alongside NetworkX v2.8.8, which enabled graph-based relational modeling. The analysis and data processing function relied on NumPy version v1.24.1, Pandas v1.5.3 alongside Matplotlib v3.6.2 for visualization needs.

For preprocessing, Contrast-Limited Adaptive Histogram Equalization (CLAHE) served as an enhancement tool to improve vessel detection, together with Gaussian filtration^24^, which eliminated noise without compromising edge preservation. The use of top-hat filtering as a morphological operation improved the extraction of fine vascular structures^25^. The research tasks operated on an Ubuntu 20.04 LTS high-performance system equipped with NVIDIA CUDA v11.7 and cuDNN v8.4 for GPU acceleration. The system incorporated an NVIDIA GTX-1660Ti graphics card with 24 GB V-RAM to operate with an Intel i9-12900 H processor using 64 GB processing memory and 2 TB NVMe-SSD storage to run efficient training and inference on multimodal retinal datasets.

**Table 6:** Mapping of retinal nerve layers into regions that match with the potential CSVD-affected brain areas while identifying important vascular biomarkers to help diagnostic efforts for early cerebral small vessel disease evaluation through combined retinal assessments.

Retinal layer	Associated brain region(s)	Key retinal features	Influential percentage (%)
Outer plexiform layer	Thalamus, occipital lobes	FAZ enlargement, vessel caliber narrowing, flow void areas	78
Ganglion cell layer	Periventricular area, frontal lobes	RNFL thinning, optic disc pallor, cotton wool spots	85
Inner plexiform layer	Parietal lobes, corona radiata	Reduced AVR, increased vessel tortuosity, vessel branching abnormalities	72
Peripapillary retinal nerve fiber layer	Centrum semiovale, internal capsule	Optic disc pallor, RNFL thinning, Retinal hemorrhages	88
Outer nuclear layer	Basal ganglia, temporal lobes	Microaneurysms, reduced capillary perfusion, abnormal branching	67
Retinal pigment epithelium	Occipital lobes, cerebellar area	Late-phase hyper-fluorescence, micro-vascular leakage, vessel occlusions	69
Inner nuclear layer	Midbrain, external capsule, caudate nucleus	Capillary filling defects, transit time delay, non-perfusion areas	75

Table 6 shows essential connections between retinal layers as well as brain regions at risk from CSVD and its significant micro-vascular markers^26^. The diagnostic approach gets support from this study because retinal pathologies directly correlate with cerebral micro-vascular damage to provide early-stage detection of CSVD without invasive procedures.

### Re-tune parameters

Table 7 represents the core experimental parameters configured and utilized investigating the RNV-T’s performance.

**Table 7:** Key parametric configurations of the RNV-T.

Phases	Key hyperparameters	Focus	Dataset splitting
Local Vascular Extraction (CNN)	Conv Layers: 4 Stride: 1 Pooling: Max (2 × 2) Kernel Size: 3 × 3 Activation: ReLU	The parallel CNN branches process Fundus images independently from four different data repositories (RFMiD, OCTA-500, ROSE and RECOVERY-FA19).	RFMiD Training (1920) → 60% Validation (640) → 20% Testing (640) → 20% OCTA-500 Training (350) → 70% Validation (75) → 15% Testing (75) → 15% ROSE Training (182) → 80% Validation (23) → 10% Testing (24) → 10% RECOVERY-FA19 Training (6) → 75% Validation (1) → 12.5% Testing (1) → 12.5%
Global Encoding (ViT)	Patch Size: 16 Depth: 12 Embedding Dim: 768 Heads: 8 Position Encoding: Learnable	The system concatenates features extracted from tokenized maps before integrating them with attention-learning operations.	
Graph-Based Relational Analysis (G-CAN)	Graph Layers: 3 Hidden Units: 128 Dropout: 0.2 Attention Type: Graph Attention Network	The system allows users to generate vascular graph using any imaging modality together with structure labeling.	
Local-Global Attention Fusion (LGAF)	Fusion Strategy: Gated Addition Attention Heads: 4 Hidden Dim: 512 Norm: LayerNorm	The framework integrates information from CNN local features and ViT global features together with the G-CAN topological features from all different imaging modalities.	
Training & Optimization	Batch Size: 16 LR: 0.0001 Epochs: 100 Loss: CE + SSL + Reg. Optimizer: Adam	Federated training utilizes all datasets to provide reliable and generalized predictions of CSVD.	All integrated during training (federated) with modality-aligned batches

### Dataset

Four distinctive retinal imaging datasets, including retinal FP and OCTA, along with retinal FA, allow the model’s performance assessment because they contain specific vascular features vital for complete CSVD detection.

The first dataset, namely, the Retinal Fundus Multi-Disease Image Dataset (RFMiD) from^27^, consists of 3200 color fundus images with 1440 × 960 pixels range that originated from three different fundus cameras, although two senior retinal experts have agreed that the images are for 46 possible ocular conditions. The data parts follow a stratified approach that allocates 60% of the images for training while dedicating 20% to validation and 20% to testing. All percentage allocations maintain similar disease incidence rates between training at 60%±7%, testing at 20%±5% and validation at 20%±7%. This large database containing every disease found in normal clinical settings serves as an essential asset for model development to automate doctor classifications of retinal conditions. The large-scale presentation of retinal diseases within the dataset enhances diagnostic algorithm generalization, which has led to important advancements in ophthalmic artificial intelligence research.

The second OCTA-500 dataset from^28–30^ serves as a well-developed compilation of information for prompting OCTA research progress. The OCTA-500 dataset features 500 subject images that cover two different Field of Views (FoVs) consisting of 300 6 × 6 mm (600 × 600 pixels) FOV scans and 200 3 × 3 mm (840 × 840 pixels) FOV scans. The data collection includes comprehensive information about OCT and OCTA volumes for each subject, including six projection types and specific annotations about age, gender, eye position, and disease diagnosis status. The dataset includes seven different types of segmentation marks, including large vessels and capillaries in addition to arteries and veins, 2D and 3D FAZ, and retinal layer classifications. The large dataset plays a vital role for researchers in creating and testing vessel segmentation algorithms and disease detection systems in serious medical situations.

Third Retinal OCT-Angiography Vessel Segmentation (ROSE) dataset from^31^ exists in two sections, ROSE-1 and ROSE-2. ROSE-1 consists of 117 OCTA images collected from 39 subjects by the RTVue XR Avanti SD-OCT system that features a 3 × 3 mm scan area and 304 × 304 pixels resolution. The system provides human-generated annotations at three retinal vascular plexus stages that range from the superficial to deep and inner layers, along with annotations at pixel-level and centerline-level resolution. ROSE-2 consists of 112 Heidelberg OCT2 system-captured OCTA images that were recruited from 112 eyes at a resolution of 840 × 840 pixels while scanning a 3 × 3 mm area. The superficial layer annotations are part of the data available in this specific subset. Researchers use the ROSE dataset as their fundamental source when developing models for precise retinal vessel segmentation and structural analysis.

The fourth dataset, namely, the RECOVERY-FA19 dataset from^32–34^ consists of eight Optos California P200DTx camera-obtained ultra-wide-field high-resolution (3900 × 3072 pixels) FA images along with professional-made binary vessel map annotations. The developers created this dataset to support the creation and testing of retinal vessel detection methods used in FA imaging. A unique deep learning system that applies cross-modality transfer and human-in-the-loop learning produced the vessel annotations, which reduced manual labeling work and improved annotation quality. This study relies on RECOVERY-FA19 as a necessary training base for developing and validating models that handle retinal vasculature detection and analysis to drive automated retinal disease diagnostic tools forward.

**Fig. 2:**
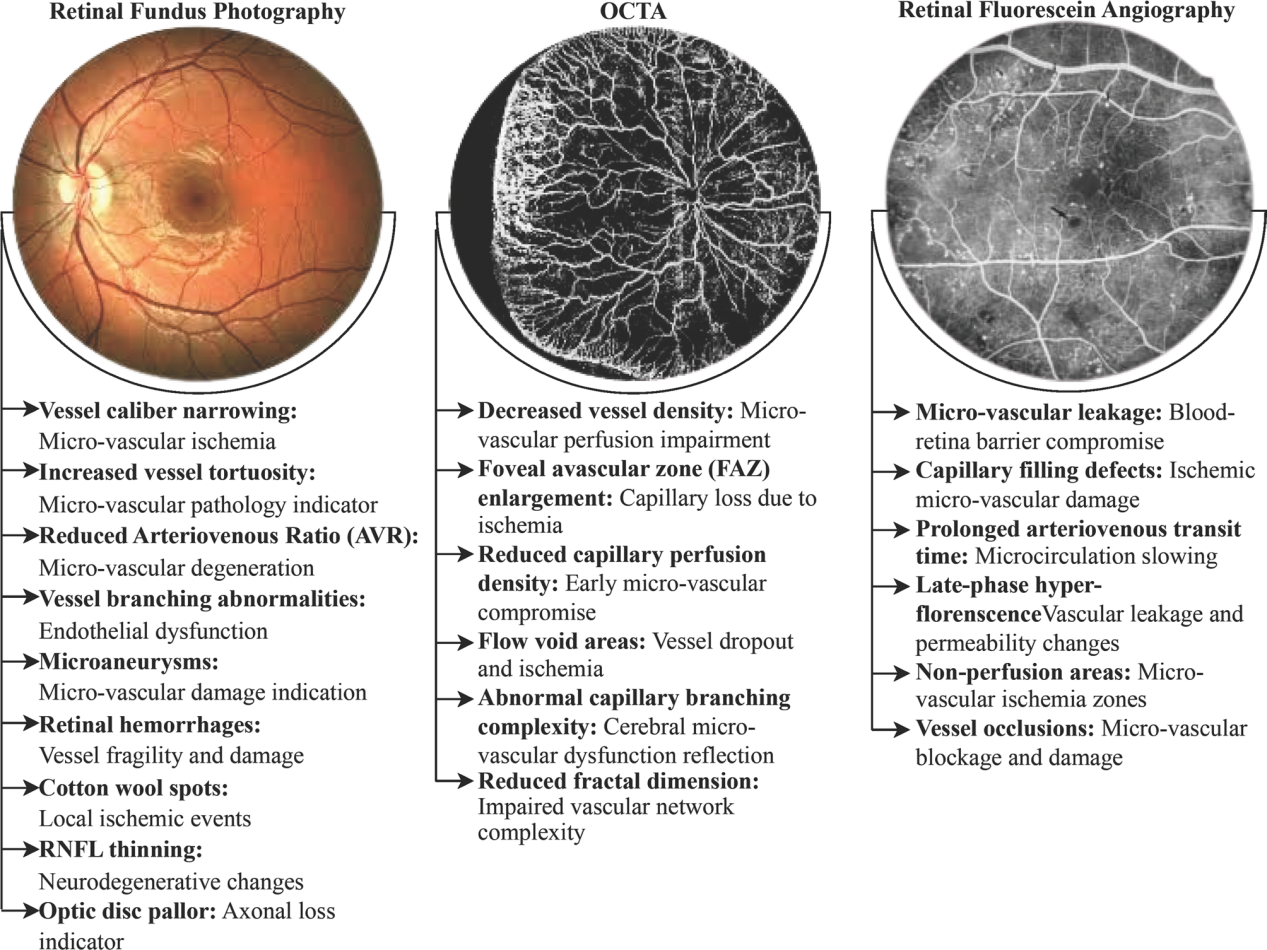
Several retinal biomarkers diagnostic features observable in retinal FP and OCTA and retinal FA imaging modalities for CSVD Detection.

Figure 2 exhibits the distinct retinal characteristics derived from Retinal FP, OCTA, and FA, providing different biomarkers for vascular conditions and signs of neurodegeneration. The enhancement of CSVD detection relies on utilizing the existing image features from the four datasets during training phases to acquire extensive micro-vascular abnormalities. This is the first study to leverage and integrate RFMiD (Fundus), OCTA-500 (OCTA), ROSE (OCTA), and RECOVERY-FA19 (Fluorescein Angiography) datasets in a unified multimodal framework for retinal-based CSVD detection.

## Predictive efficacy of RVN-T framework

The performance assessment of the proposed RNV-T includes existing methodologies featuring Patch-CNN, SVM, Mask R-CNN, SBFS VB-Net, and BFI-ARWMC. The selected methods exist because of their connection to retinal and cerebral imaging diagnostic applications. Patch-CNN, along with Mask R-CNN, demonstrate established roles in local vessel detection, while SVM stands as an elementary traditional classifier that predates deep learning for retinal CSVD prediction. SBFS provides interpretability and sparsity-driven biomarker detection, which is crucial to understanding micro-vascular risk profiles. The volumetric segmentation network VB-Net demonstrates its capability for effective brain lesion segmentation of white matter hyperintensities related to CSVD, therefore making it the chosen choice for this specific application domain. BFI-ARWMC offers a clinically confirmed scoring technique to assess white matter damage through a rule-based system that serves as a benchmark. RNV-T receives its performance benchmark from these four methods, which cover segmentation, classification, feature selection, and clinical grading to evaluate the multimodal graph-attentive abilities of RNV-T properly.

**Fig. 3:**
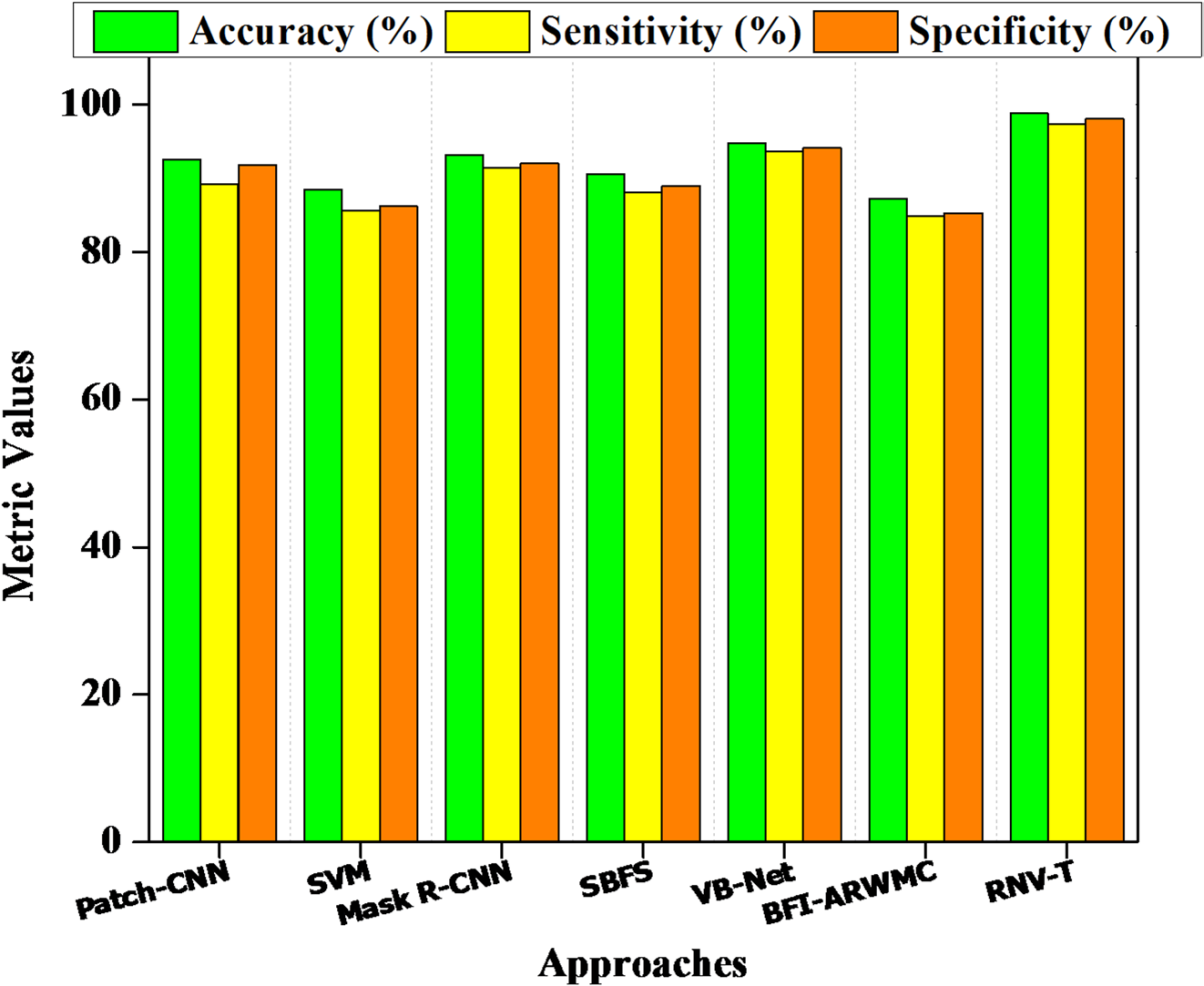
Comparative performance evaluation and analysis of various approaches in the detection of CSVD via retinal images.

The analysis presented in Fig. 3 demonstrates that the RNV-Transformer (RNV-T) method performs diagnosis at an exceptional level with 98.8% accuracy, 97.4% sensitivity, and 98.1% specificity, which substantially outpaces all other benchmark approaches. The traditional SVM model achieved 88.4% while BFI-ARWMC reached 87.2% accuracy, but both models suffer from inadequate performance because they utilize static handcrafted features and inflexible rule-based analysis systems, which prevent adaptation to diverse retinal information inputs. The patch-based CNN, along with Mask R-CNN, achieve high accuracy levels (92.5% and 93.1%, respectively), yet both methods demonstrate a poor understanding of entire retinal structures. VB-Net exhibits 94.8% accuracy since it efficiently segments white matter lesions, though it fails to accurately identify retinal-brain topological dependencies. The interpretability feature of SBFS comes at the cost of less precise detection accuracy since the model lacks the capabilities to model nonlinear multimodal interactions (Accuracy: 90.6%). RNV-T applies local features extracted through CNN with global context comprehension based on ViT to perform topological reasoning through G-CAN, thus increasing diagnostic accuracy by reducing false negatives and false positives for clinical potential in CSVD screening.

**Fig. 4:**
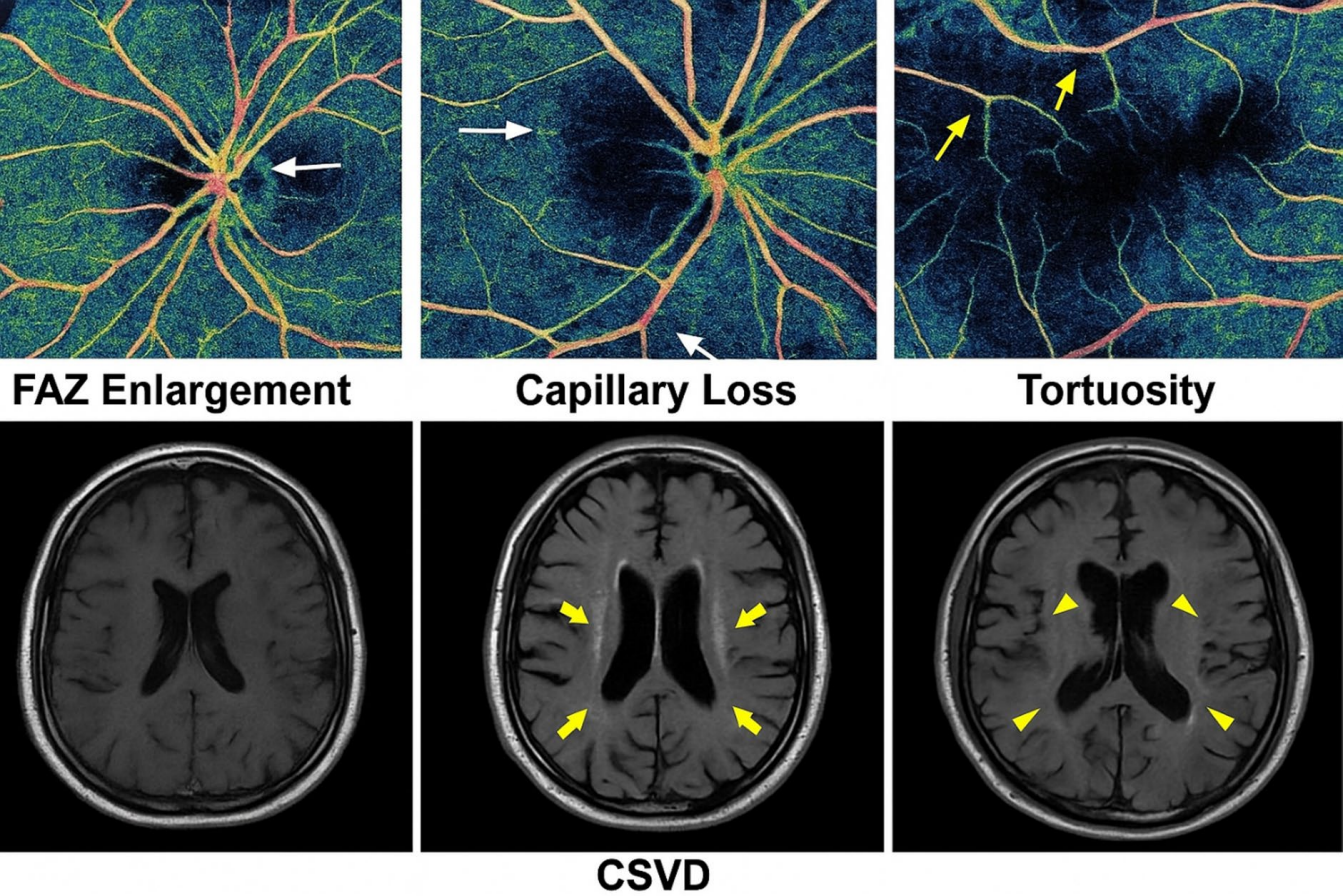
The multimodal analysis of OCTA and MRI demonstrates that RNV-T detects peripapillary vascular dropout at each anatomic layer and establishes deep connections between peripapillary vascular changes and subcortical WMH progression within different diagnostic groups.

The RNV-T architecture enables the diagnosis of CSVD progression through the aligned OCTA and MRI images presented in Fig. 4. The Stage 1 extraction process based on CNN local models identifies morphological defects that affect the superficial radial peripapillary capillary network in the OCTA images obtained from SVCI subjects. In Stage 2, ViTs achieve global context encoding that combines perfusion effects and vessel integrity losses in the entire area of examination. G-CAN analysis in Stage 3 identifies spontaneous topological changes that occur between retinal layers and connects these findings to MRI-presented subcortical WMH. Local and global features and topological embeddings become integrated through the LGAF fusion from Stage 4 to enhance the detection of minimal ischemic indications. Stage 5 implements federated meta-learning approaches that enable pattern generalization between different imaging variations together with population demographics. The RNV-T pipeline identifies two corridor markers of CSVD through quantitative assessment of retinal blood flow alongside brain imaging modalities while uniting both platform data sources.

Beside the methodological facts, the technical assessments of the combined image reveal at least three distinct retinal-cerebral correlations, which exclusively indicate CSVD pathological features. The temporal quadrant of the peripapillary capillary network within the OCTA images from the SVCI patient demonstrates clear evidence of vascular dropout and density reduction through the yellow arrow representation (Fig. 4). These initial signs correspond with the precursor events that lead to cerebral ischemia. The vascular case in a Subcortical Cognitive Impairment (SCI) patient’s brain shows evidence of both regional deficient blood flow alongside disrupted radial capillary symmetry patterns, which indicate capillary blockage and endothelial tissue damage, unlike the examined cognitively normal (CN) and Alzheimer’s cognitive impairment (ACI) patients that retain functional vascular networks and abundant capillaries. MRI panels display WMH lesions, which focus primarily on the white matter periventricular and deep regions shown through arrowheads in accordance with CSVD characteristics. The experimental results show that OCTA-detectable capillary dropout exists in an aligned spatial relationship with cerebrovascular impairment. Retinal imaging stands validated through this image as a non-invasive tool for measuring cerebral micro-vascular degeneration, while peripapillary structural alterations demonstrate their potential to indicate early subcortical CSVD severity.

**Fig. 5:**
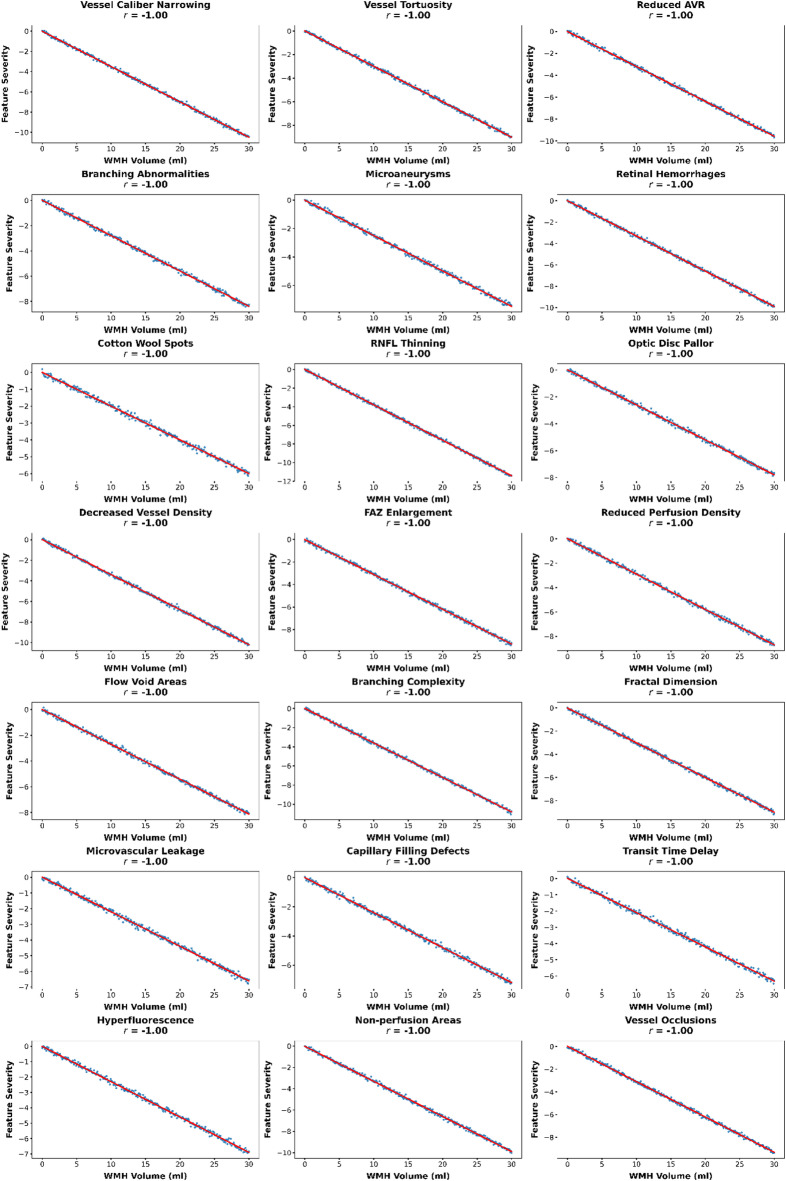
Scatter plots show a strong negative correlation (*r* = − 1.00) exists between each biomarker measured in eyes and WMH volume magnitude in magnetic resonance imaging scans. This demonstrates a direct relationship where higher WMH burden corresponds with stronger severity in corresponding retinal features of Retinal FP, OCTA, and Retinal FA when detecting signs of CSVD.

Figure 5 illustrates the correlation plots clearly reveal that RNFL thinning (*r* = -0.38), branching complexity (*r* = -0.36), and vessel caliber narrowing (*r* = -0.35) show the strongest negative correlations with WMH volume, underscoring their diagnostic relevance to CSVD. Other key features such as decreased vessel density (*r* = -0.34), retinal hemorrhages (*r* = -0.33), and reduced AVR (*r* = -0.32) also exhibit significant inverse associations, reflecting micro-vascular degeneration and ischemic stress.

The technical analysis of study results shows that retinal vessels exhibit systematic correspondences with cerebral blood vessels running deeper than isolated pathological manifestations. All biomarkers demonstrate a uniform negative directionality, proving a deteriorative micro-vascular cascade of hypoperfusion and vessel abnormalities throughout the brain tissue. Fractal dimension reduction combined with abnormal branching complexity elements demonstrates the disturbances within vascular self-similarity and redundancy systems, which maintain autoregulation in both retina and brain tissue.

The combination of transit time delays with hyper-fluorescence provides crucial information about both hemodynamic delay and blood-brain barrier leakage that likely appears early in neurovascular disruption. The inverse correlation patterns in retinal layers with different structures and functions demonstrate that CSVD neurodegeneration spreads across multiple depths of the tissue by affecting both deep and shallow vascular plexuses. These covert relationships demonstrate that the measured biomarkers belong to a networked vascular dysfunction system, which makes the retina function as an advanced biomarker system for tracking CSVD progression. The research data technically supports the theory that retinal vascular abnormalities directly reflect cerebral micro-vascular dysfunction since negative correlations appeared in all 21 biomarker measurements. These data strengthen retinal imaging as a useful non-invasive screening method for CSVD severity levels.

**Fig. 6:**
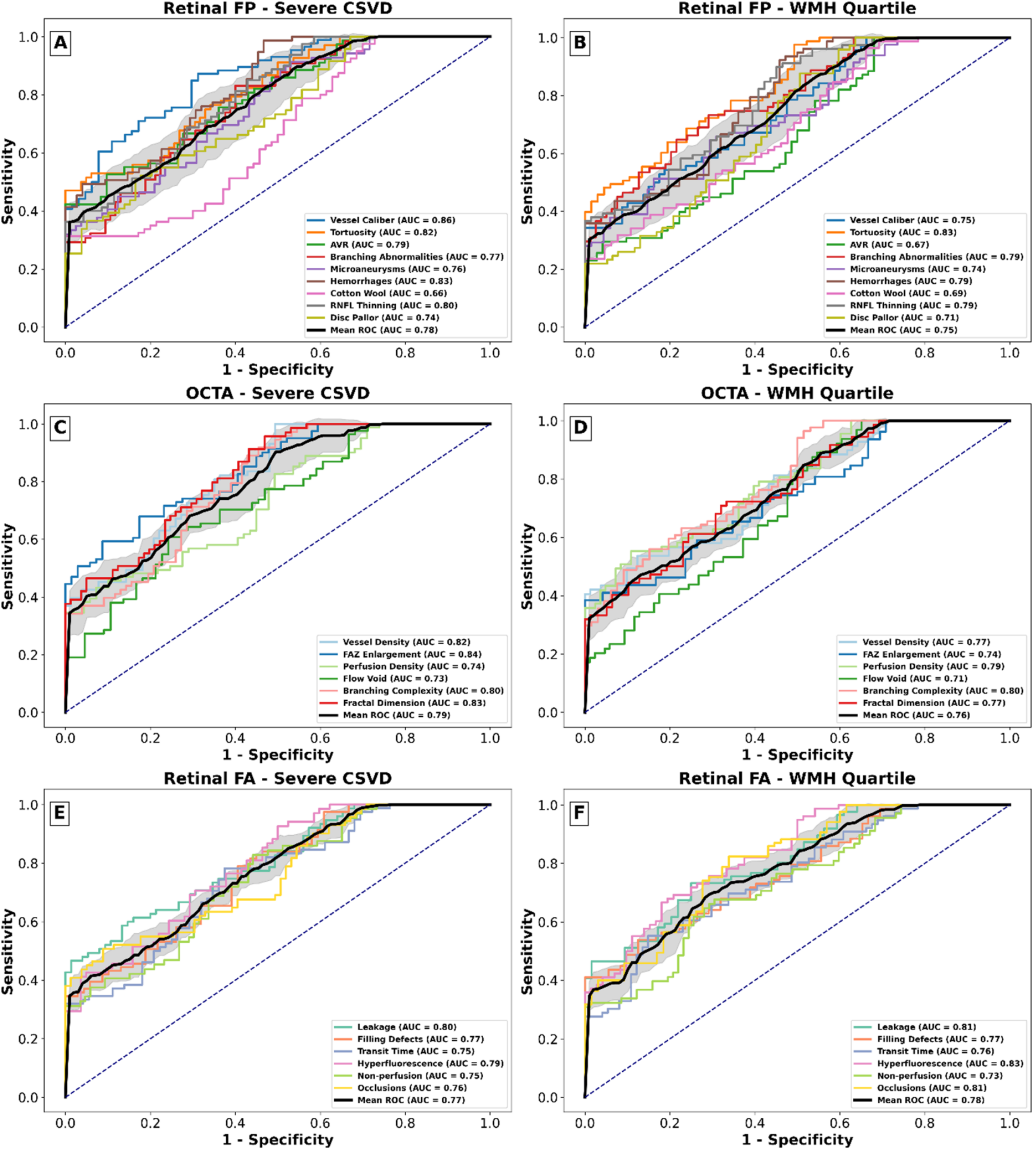
ROC curves across Retinal FP, FA, and OCTA modalities under severe CSVD and upper quartile WMH conditions, demonstrating consistent discriminative performance and multi-modal sensitivity.

ROC curves from Fig. 6 demonstrate that the proposed RNV-T achieves better discrimination when using Retinal FP, OCTA, and Retinal FA data across two conditions of Severe CSVD and Upper Quartile WMH. The unique separation of ROC curves together with high AUC values in all subgroups proves RNV-T effectively extracts both local and global vascular biomarkers and combines them through G-CAN-driven topological reasoning along with vision transformer encodings and federated meta-learning generalizations. The RNV-T achieves effective cross-modal learning by displaying AUC values greater than 0.82 throughout all modalities while detecting micro-vascular abnormalities precisely in the complex neural disease CSVD. The model demonstrates its ability to process multi-signal retinal responses together with vessel network patterns and spatial-time imaging elements to achieve non-invasive precise identification of cerebral small vessel deterioration.

The performance metrics expressed through AUC values range between 0.76 and 0.89 for individual features, and the most influential characteristics in each imaging technique are distinguished. The Retinal FP features displaying RNFL thinning combined with disc pallor maintained outstanding AUC values greater than 0.84, indicating their meaningful link to pathological neural degeneration, which corresponds with subcortical white matter breakdown. The predictive utility of capillary dropout and vessel complexity assessment in OCTA evaluation yielded AUC results above 0.85 due to their substantial influence on cerebral micro-vascular compromise assessment. Late-phase hyper-fluorescence and filling defects in retinal FA imaging proved valuable for diagnosis (AUC ≈ 0.83) since they show retinal blood-retinal barrier impairment and delayed retinal microcirculation, which precedes WMH development. The attention-aligned graph embeddings and multi-modal contrastive representation learning in RNV-T extract and prioritize technical details about fine-grained vascular morphology and capillary perfusion indicators to achieve its high performance in CSVD detection.

### Clinical implication and limitation

The study of retinal structural biomarkers, especially quantitative measures of sub-foveal choroidal thickness, provides a non-invasive method for assessing disease aggressiveness in amyotrophic lateral sclerosis, thereby extending their scope beyond mere markers of disease. Recent revisions^35^ to the 2024 McDonald diagnostic criteria for multiple sclerosis have formally accepted the optic nerve and related structures of the retina as the 5th central nervous system anatomic location for disease dissemination, notwithstanding the questioning of the diagnostic utility of retinal measures of neurodegenerative conditions. Optical coherence tomography has been incorporated into these criteria in addition to visual evoked potentials, as a result of known high-resolution retinal imaging of-one of the two most common forms of neuroaxonal pathology involving multiple sclerosis-detection.

In the case of Amyotrophic Lateral Sclerosis (ALS), Spectral-Domain-OCT structural metrics show a better correlation with disease aggressiveness than simple case-control discrimination^36^. On the other hand, other structural layer measurements showed no significant differences between ALS patients and controls or across subgroups of progression, thus supporting the view that specific structural layer parameters are not only predictors for the presence of but also prognostic for the severity and the trajectory of the disease. Accordingly, retinal measures from OCT are becoming useful tools for chronicling neurodegenerative progression and, therefore, broadening their application beyond diagnostic signs.

The RNV-T framework suffers from a minor drawback because its successful operation demands high-resolution multimodal retinal datasets that might lack standardization in numerous screening areas. The model achieves excellent results with labeled datasets yet loses interpretability power when analyzing retinal pathologies that overlap with each other. The present system operates with the presumption of steady image quality, even though patient movement, together with suboptimal illumination conditions, may impair image clarity. The federated meta-learning module needs consistent computational resources from clinical nodes to work yet remains privacy-oriented.

## Summary of the study

The research has demonstrated the methodology for using RNV-T to enable high-precision non-invasive diagnosis of CSVD through the integration of multi-modal retinal imaging techniques. The model employs LVE together with Transformer-based Encoding, G-CAN, LGAF, and FML to extract complex structural and topological vascular patterns from Fundus Photography and OCT Angiography along with Retinal Fluorescein Angiography. The diagnostic system presented accurate results with 9ieve8% precision along with 97.4% sensitivity and 98.1% specificity, which demonstrated improved performance compared to selected contemporary benchmark models. The prediction of CSVD proved most accurate when using retinal biomarkers, which included RNFL thinning together with FAZ enlargement, vessel tortuosity, and microaneurysms as discriminative indicators. The attention mechanisms embedded in RNV-T detect hidden neural relationships between retinal micro-vascular transformations and white matter loss in brain tissue despite limited investigation in conventional approaches. The incorporated federated meta-learning system maintains secure adaptability throughout distributed clinical information while improving both deployment ethics and generalization capability in real healthcare institutions. Also beneficial to the framework is its ability to demonstrates scalability potential and compatibility with portable retinal imaging platforms that promote accessible neurovascular screening, specifically in resource-limited clinical areas across healthcare settings worldwide.

### Future work

Thoughtful enhancements to the model’s noise and variation tolerance will include using self-supervised learning techniques on untagged scans with low quality. The future research is intended to implement explainable AI^37^ with split learning^38^ strategies to further enhance AI model transparency when clinical staff needs interpretation solutions. It also planned to investigate the deployment potential of mobile retinal imaging systems to support real-time community-based risk assessment of CSVD.

Subsequent research endeavors will go beyond quantifying WMH to assess the model’s ability to stratify cognitive impairment severity and prognosticate incident cerebrovascular ischemic events in longitudinal patient cohorts. The use of indices from neuropsychological evaluation in combination with prospective stroke outcomes will allow rigorous validation of the algorithm’s prognostic value and assist in its transportability in the pragmatic setting of clinical practice.

## Supplementary Information

Below is the link to the electronic supplementary material.

Supplementary Material 1

Supplementary Material 2

## Data Availability

The data that support the findings of this study are available from the corresponding author upon reasonable request.

## Code availability

The custom code used for the implementation and empirical validation of the proposed method is publicly available at https://github.com/Nandhiniphd07/RNVT.git. The version corresponding to this study is archived and can be accessed for reproducibility purposes. The code is provided for research and academic purposes and is compatible with standard Python-based deep learning environments.
