# Supplementary material for: Deep learning for early detection of cerebral small vessel disease using self-supervised graph embeddings and retinal image analysis
Source: Sci Rep. 2026 Apr 15;16:17579. doi: 10.1038/s41598-026-48421-6 (PMC13243560; doi:10.1038/s41598-026-48421-6)
Supplement: Supplementary file 1 — Supplementary Material 1 [file 41598_2026_48421_MOESM1_ESM.pdf]

Gmail

Nandhini S <nandhiniphd07@gmail.com>

✅ Access request for 'ROSE: Retinal OCT-Angiography segmentation dataset' was accepted.

Zenodo <noreply@zenodo.org> Tue, Feb 24, 2026 at 4:32 PM
To: nandhiniphd07@gmail.com

Your access request for 'ROSE: Retinal OCT-Angiography segmentation dataset' was accepted.

Access the record
