# Supplementary material for: Deep learning for early detection of cerebral small vessel disease using self-supervised graph embeddings and retinal image analysis
Source: Sci Rep. 2026 Apr 15;16:17579. doi: 10.1038/s41598-026-48421-6 (PMC13243560; doi:10.1038/s41598-026-48421-6)
Supplement: Supplementary file 2 — Supplementary Material 2 [file 41598_2026_48421_MOESM2_ESM.pdf]

WITH IEEE

IEEE

You Can Achieve Great Things.

Thank You for Your Membership!

You are a member of the
Madras Section

Below is a digital version of your membership card for easy access to your membership information. You can also access this in your IEEE profile or in the IEEE app anytime!

Member **2025**

VANITHA K

Member # 99431026
Madras Section

IEEE Member for 3 years
Valid through 31 December 2025

Kathleen A. Kramer
2025 IEEE President

IEEE

For membership information visit **ieee.org/start**

IEEE is a New York not-for-profit corporation. This membership card is for the named member only and is non-transferable. All members of the IEEE shall be governed by IEEE's Constitution, Bylaws, Policies, and Code of Ethics.

IEEE Contact Center:
ieee.org/contactcenter
+1 800 678 4333
USA/Canada
+1 732 981 0060
Worldwide

3 Park Avenue, 17th Floor
New York, NY 10016-5997 USA

IEEE
Advancing Technology for Humanity

Make the Most of Your Membership.

Local IEEE Section
Get involved with colleagues at your local IEEE Section, who can help connect you to professionals who can advance your goals.

Technical Publications
Take advantage of discounts and access to cutting-edge journals, magazines, and other publications.

Career Opportunities
Drive your career goals forward with online learning, job listings, a consultant's network, and more.

Professional Network
Build a professional network from the wealth of diversity, expertise, and connections found within IEEE.

IEEE.tv
Watch live streaming coverage from IEEE events and IEEE conferences and learn from thousands of programs about today's technologies, and more.

IEEE Conferences
IEEE members receive deeply discounted access to technical seminars, IEEE conferences, and conference papers.

Learn about these and all IEEE member benefits at **ieee.org/benefits**
